# An indirect watermark hiding in discrete cosine transform–singular value decomposition domain for copyright protection

**DOI:** 10.1098/rsos.170326

**Published:** 2017-06-28

**Authors:** Soumitra Roy, Arup Kumar Pal

**Affiliations:** 1 Department of Computer Science and Engineering, Dr. B. C. Roy Engineering College, Durgapur, West Bengal 713206, India; 2 Department of Computer Science and Engineering, Indian Institute of Technology (Indian School of Mines), Dhanbad, Jharkhand 826004, India

**Keywords:** Arnold scrambling, discrete cosine transform, imperceptibility, robust watermarking, singular value decomposition

## Abstract

Digital image watermarking has emerged as a promising solution for copyright protection. In this paper, a discrete cosine transform (DCT) and singular value decomposition (SVD) based hybrid robust image watermarking method using Arnold scrambling is proposed and simulated to protect the copyright of natural images. In this proposed scheme, before embedding, watermark is scrambled with Arnold scrambling. Then, the greyscale cover image and encrypted watermark logo are decomposed into non-overlapping blocks and subsequently some selected image blocks are transformed into the DCT domain for inserting the watermark blocks permanently. For better imperceptibility and effectiveness, in this proposed algorithm, watermark image blocks are embedded into singular values of selected blocks by multiplying with a feasible scaling factor. Simulation result demonstrates that robustness is achieved by recovering satisfactory watermark data from the reconstructed cover image after applying common geometric transformation attacks (such as rotation, flip operation, cropping, scaling, shearing and deletion of lines or columns operation), common enhancement technique attacks (such as low-pass filtering, histogram equalization, sharpening, gamma correction, noise addition) and jpeg compression attacks.

## 1. Introduction

Nowadays with the advancement of high-speed communication network and personal computers, access, transmission, saving and distribution of digital data (such as images, video, audio and text) are becoming less complex and very much time and cost effective. But in today's forgery world, modification and perfect replication of digital data with the help of easy-to-use software and cheap digital materials like video cameras is turning out to be common practice for illegal uses. Internet is used in an illicit manner to distribute this modified and replicated illegitimate digital content. Therefore, authentication, integrity violation and copyright protection issues have been developed as a serious threat for this transmitted digital information. To avoid this type of security threat, sometimes extra digital data-related information is embedded into digital data in imperceptible way (termed as digital watermarking). Over the last few years, digital watermarking technology has been proposed as a better solution than cryptographic [1] and steganographic processes to overcome this authentication and copyright protection problem. In digital image watermarking method, certain information [2] (such as signature and logo ID number) uniquely related to the owner or distributor is permanently embedded into digital data so that it can be later detected or extracted for the verification of rightful ownership. The embedded watermark should still be detectable, while the perceptual quality of digital data remains to an acceptable level. The important parameters for digital image watermarking scheme are [3] *imperceptibility*, *robustness*, *embedding capacity* and *readily embeddable and extractable watermark data.* Among these characteristics, imperceptibility property is inversely proportional to the watermark embedding capacity. But, in general convention, robustness is improved with the increment of embedding capacity. So there should be some settlement between these three properties to design an effective watermarking scheme.

Depending on the various properties, watermarking technique can be divided into various ways [1]. According to the human perception, watermarking can be divided into visible and non-visible watermarking. In visible watermarking, transparent watermark is present in the cover image. But preservation of the visual quality of watermark object and imperceptibility of watermark logo (by human perception) is the main concern in the non-visible watermarking technique. Watermarking schemes can also be divided into three schemes as blind, semi-blind and non-blind watermarking schemes. In blind watermarking scheme, neither original data nor watermark data are needed in detection process; whereas in semi-blind scheme, watermark data and some other side information are needed in detection process; while in non-blind watermarking scheme, original data and sometimes watermark data are needed in detection procedure. Owing to the need of the original data at the receiver side, non-blind scheme suffers from security compromises and practical application constraints [4]. In the early days, non-blind schemes were more robust than blind schemes, but in today's scenario, performance criteria of blind-based watermarking scheme are almost identical to those of non-blind schemes. Depending on its application, watermarking schemes can be classified into robust watermarking schemes and fragile watermarking schemes. Robust digital watermarking techniques are applied for copyright protection. In robust digital watermarking techniques, embedded watermark information almost preserves the image perceptual quality while resisting all types of attacks or changes to the watermarked image. Fragile watermarking schemes are widely used for data authentication and temper detection. In fragile watermarking schemes, the aim is to embed a watermark that should be alterable under common signal processing attacks. A semi-fragile watermarking scheme, which is designed according to the integrity property of cover object, is the mixture of robust and fragile watermarking scheme. Similar to the robust watermarking technique, semi-fragile watermarking can bear some kind of modification to the watermarked object by legitimate distortion while, like fragile one, it can detect the region of the object that is modified by illegitimate distortion and distinguish them from the authentic or unaltered region.

Recently, researchers have proposed so many effective singular value decomposition (SVD) based watermarking schemes due to their attractive properties, and [5] geometric features from an image are easily obtained by SVD mathematical techniques. The main features of SVD are described in detail in §2.2. Basically, in SVD based watermarking scheme, SVs of an image are modified to embed watermark or watermark SVs. Liu & Tan [6] proposed a pure SVD based blind watermarking scheme. With the aim to increase the invisibility and capacity of SVD based watermarking scheme, Chung *et al.* [7] proposed two notes in which they modified the coefficients in both U and V components after SVD transformation. For further improvement of method in [7], Fan *et al.* [8] modified only the first column of U and V matrices after SVD transformation.

But researchers found that there are some serious drawbacks in SVD-based singular value decomposition (SVD) based algorithm: (i) *lower invisibility* [9] in watermark image because one singular value modification alters all values in a pixel matrix; (ii) existence of *false positive detection problem* [10] in most SVD based watermarking methods; and (iii) alteration of the SVs of the cover image with the watermark image may create one *diagonal line* in [11] the recovered watermark. To achieve better robustness and high imperceptibility, recently researchers have developed their watermarking scheme using two or three transform domain techniques. These hybrid domain techniques provide better results than their single counterpart. In addition to this, the application of SVD mechanism in one single image requires huge computation [12]. So hybrid domain watermarking scheme is becoming more effective than pure SVD. Makbol & Khoo [12] present a new hybrid image watermarking scheme based on the redundant discrete wavelet transform (RDWT) and SVD. But, it is shown by Ling *et al.* [13] that the robust blind image watermarking scheme based on RDWT and SVD [12] has a fundamental flaw in its design that undermines the security of its scheme against the false positive problem. A non-blind DCT-SVD based hybrid domain watermarking is presented in [14]. In this technique, discrete cosine transform (DCT) is applied to the cover image at first, and then coefficients are scanned in zigzag order. Then SVs of the cover image are modified with the SVs of DCT transformed visual watermark. Its main disadvantage lies in its computational value. A DWT-SVD based watermarking scheme is proposed in [15]. After decomposing the cover image into four sub-bands, SVs of each sub-band are modified by SVs of watermark data in this technique. A DWT-SVD based watermarking technique is proposed in [16]. An RDWT and SVD based hybrid watermarking scheme is proposed in [17]. Rastegar *et al.* [18] proposed hybrid domain watermarking technique using SVD and radon transform. In [19], a new hybrid, secure and robust image watermarking scheme based on the integer wavelet transform (IWT) and SVD is proposed. In this work, authors raise the issue of the false positive problem for most of the SVD based watermarking schemes. Two reasons for this problem are also discussed in this paper:

(i) Reason 1: For watermark embedding, the modification of the SVs of the host with the SVs of the watermark.
(ii) Reason 2: Use of the following equation in embedding process:
  1.1 $$S+\alpha \times W=U_{W}S_{W}V_{W}^{T}.$$

In watermark embedding step, embed the watermark into the SVs of cover image by multiplying with a scaling factor of *α*. In the next step, apply SVD to obtain $U_{W}$, $V_{W}$ and $V_{W}^{T}$ matrices and keep $U_{W}$ and $V_{W}^{T}$ as secret keys for extraction purpose (as *U* and *V* preserve major information of an image). Therefore, illegitimate user can allege ownership using counterfeited $U_{C}$ and $V_{C}^{T}$.

Modern researchers suggest lots of solutions for the above stated security concerns. In [19], it has been solved by adopting a digital signature into the watermarked image. In another research work, Loukhaoukha *et al.* [20] encrypted the watermark before inserting it into the cover object. This encryption policy is adopted in this proposed method. The literature survey suggests that robustness of image watermarking scheme can be improved with the proper combination of SVD and suitable transform domain technique. To improve the effectiveness of watermarking scheme, in this proposed hybrid domain technique SVD is combined with block based DCT, and SVs of an image are modified with Arnold scrambled watermark data and not with the watermark SVs.

After this introductory section, the rest of the paper is organized as follows. Preliminaries of the DCT and SVD and Arnold scrambling are presented in §2. The proposed watermark embedding and extraction algorithms are described in §3. Experimental results are furnished in §4. Finally, conclusions are drawn in §5.

## 2. Preliminaries

In the proposed robust image watermarking scheme, the greyscale encrypted watermark information is embedded in SVD domain. To make the scheme robust, watermark embedding process is developed in the DCT-SVD based hybrid domain. So in this section, DCT and SVD and Arnold scrambling are briefly explained.

### 2.1. Block based discrete cosine transform

DCT [21] is one of the popular and widely used signal decompositions and compression techniques that transform a signal from spatial domain representation into a spectral representation with an inherent ability to exhibit excellent energy compaction for the signal or image. It basically transforms the signal as a sum of sinusoids of varying magnitudes and frequencies. This transformation is used for transferring pixel values of the image from one domain to the other. DCT transformation consists of one DC coefficient and AC coefficients. Most of the information of transformed image stored in few low level frequency coefficients lies on the upper top left corner of the image. DC coefficient is the average of the pixels of the image and AC coefficients contain the significant information of the image, but less than that of DC component. In block based DCT, the input image, *f* of size *M* × *N* is decomposed into non-overlapping blocks of size *m* × *n* and then each block *f*_b_ is transformed into corresponding DCT coefficients according to the following equation:

2.1 $$F_{b}(u,v)=\alpha (u)\alpha (v)\sum_{x=0}^{m-1}\sum_{y=0}^{n-1}f_{b}(x,y)\cos\left[\frac{(2x+1)u\pi }{2m}\right]\cos\left[\frac{(2y+1)v\pi }{2n}\right],$$

where

2.2 $$\begin{array}{rl} \alpha (u) & =\left\{\begin{array}{cc} \sqrt{\frac{1}{m}}, & u=0\\ \sqrt{\frac{2}{m}}, & \text{otherwise} \end{array}\right\}\\ \text{and}\;\alpha (v) & =\left\{\begin{array}{cc} \sqrt{\frac{1}{n}}, & v=0\\ \sqrt{\frac{2}{n}}, & \text{otherwise} \end{array}\right\}. \end{array}\}$$

Sub-image is reconstructed from the transformed sub-image block *F*_b_(*u*,*v*) by applying

2.3 $$f_{b}(x,y)=\alpha (u)\alpha (v)\sum_{u=0}^{m-1}\sum_{v=0}^{n-1}F_{b}(u,v)\cos\left[\frac{(2x+1)u\pi }{2m}\right]\cos\left[\frac{(2y+1)v\pi }{2n}\right],$$

for *x* = 0,1,2,…,*m* *−* 1 and *y* = 0,1,2,…,*n* *−* 1 and *α* is defined as in equation (2.2).

**Figure 1.**
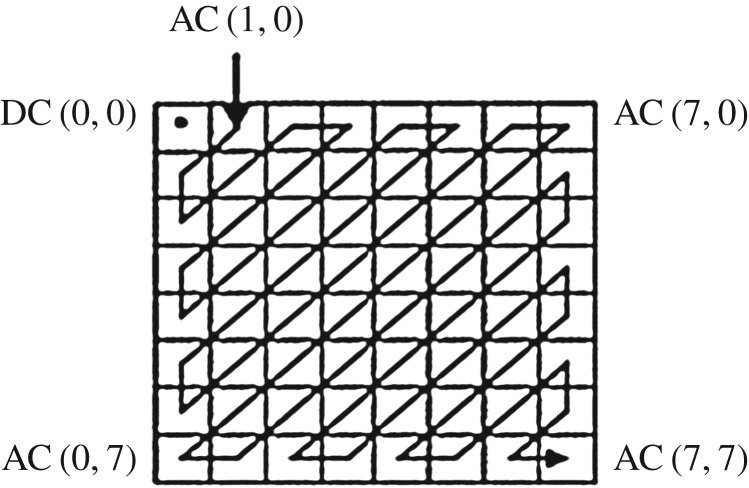
DCT's DC and AC coefficients of 8 × 8 image subpart in Zigzag order.

In block based DCT, significant information of the image decreases while traversing from one coefficient to another coefficient in zigzag order. Therefore, few upper coefficients of zigzag order are sufficient to represent an approximate image. Actually block based DCT produces three different frequency bands, namely high, middle and low frequency bands, as signal energies. In general, modification of low frequency band distorted the perceptual quality of the image as it contains maximum image information, while high frequency band can be removed by compression. That is why DCT based watermarking scheme is actually developed on using middle band frequency as it is less perceptible on modification. The top upper left DCT component of block based DCT image is *F*(0, 0), the average intensity of the image, and also known as the DC coefficient or the energy of the image. The other components of the DCT image are called AC coefficients with different low level values. In this proposed scheme, every coefficient of selected block is modified (figure 1).

### 2.2. Singular value decomposition

In the linear algebra, SVD is a stable and reliable orthogonal matrix decomposition method which splits the system optimally in such a way that each linearly independent set persists with its own energy contribution [22]. In image processing, digital Image *A* of size *n* × *n* can be decomposed by its SVD as follows:

2.4 $$A=USV^{T}$$

2.5 $$=\left|\begin{array}{cccc} u_{11} & u_{12} & \cdots & u_{1n}\\ u_{21} & u_{22} & \cdots & u_{2n}\\ \vdots & \vdots & \ddots & \vdots \\ u_{n1} & un_{2} & \cdots & u_{nn} \end{array}\right|\left|\begin{array}{cccc} \sigma_{11} & 0 & \cdots & 0\\ 0 & \sigma_{22} & \cdots & 0\\ \vdots & \vdots & \ddots & \vdots \\ 0 & 0 & \cdots & \sigma_{nn} \end{array}\right|{\left|\begin{array}{cccc} v_{11} & v_{12} & \cdots & v_{1n}\\ v_{21} & v_{22} & \cdots & v_{2n}\\ \vdots & \vdots & \ddots & \vdots \\ v_{n1} & v_{n2} & \cdots & v_{nn} \end{array}\right|}^{T}.$$

where *U*, left singular matrix, is an *n* × *n* matrix with orthogonal columns, i.e. *U*^T^*U* = *I_n_*, where *I_n_* is the *n* × *n* identity matrix. *V*, right singular matrix, is an *n* × *n* orthogonal matrix, i.e. *V*^T^ = *V*^−1^. *S* is an *n* × *n* diagonal matrix with non-negative SVs entries and these singular values are in sorted order or satisfying the condition $\sigma_{11}>\sigma_{22}>\cdots >\sigma_{nn}$. The superscript T denotes the transpose of the matrix.

Owing to their attractive properties, SVD factorization methods efficiently compress an image matrix to a smaller sized matrix with almost identical representation of its energy compaction properties [23]. Main features of SVD based methods to apply in watermarking schemes are [23,24] as follows:

(i) The SVs of an image have a very good stability. Let $X,Y\in R^{m\times n}$and their corresponding SVs are $\sigma_{1},\sigma_{2},\sigma_{3},\ldots ,\sigma_{n}$ and $\tau_{1},\tau_{2},\tau_{3},\ldots ,\tau_{n}$, respectively, and they satisfied the condition $|\sigma_{i}-\tau_{i}|\leq ∥X-Y_{2}∥$, i.e. SVs of an image have so much stability that the addition of disturbance to the SVs is not greater than 2-norm of disturbance matrix. So, when a small perturbation is added to an image, its SVs do not change significantly;
(ii) The SVs of an image exhibit proportionality property. The SVs of $X(\sigma_{1},\sigma_{2},\sigma_{3},\ldots ,\sigma_{n})$ and the SVs of $kX(\sigma_{1},\sigma_{2},\sigma_{3},\ldots ,\sigma_{n})$ are related as $∥k∥(\sigma_{1},\sigma_{2},\sigma_{3},\ldots ,\sigma_{n})=(\sigma_{1},\sigma_{2},\sigma_{3},\ldots .,\sigma_{n})$ means proportion invariance of SV must depend on the standardization of SV.
(iii) The SVs of an image show transpose property. *X* and its transposed $X^{T}$ have same number of SVs.
(iv) *X* and its flipped versions about vertical axis and about horizontal axis have the same number of non-zero SVs.
(v) *X* and its rotated version (by an arbitrary angle) have the same number of non-zero SVs.
(vi) Suppose, $X\in R^{m\times n}$ has the SVs $\sigma_{1},\sigma_{2},\sigma_{3},\ldots ,\sigma_{n}$, then its scaled version $X^{s}$ (*s* = scaling factor) has the $\sigma_{i}\times \sqrt{\left(L_{r}L_{c}\right)}$ where *i* ranges from 1,2,…,*n*. and *L*_r_ and *L*_c_ are the scaling factors of rows and columns.
(vii) Both the matrix *A* and its translated counterpart *A*^T^ have the same non-zero singular values.
(viii) For SVD decomposition, the size of matrices can be either square or rectangle.
(ix) Each singular value specifies the luminance of an image layer, while the corresponding pair of singular vectors specifies the geometry of the image.

### 2.3. Arnold scrambling

In order to expand the robustness of the algorithm and provide extra security to the embedded watermark, Arnold scrambling is employed in the pre-processing step of the proposed method. The classical Arnold scrambling jumbles up the pixel positions of the host image to generate a chaotic image and thus takes the responsibility to act as a secondary encryption technique. Eventually, the watermark is shared out in all space of the host image as space locations of watermark pixels are disturbed by scrambling method. This muddled watermark cannot be recovered without proper information about the scrambling algorithm, even if the attacker successfully extracts the watermark from cover image. Hence, scrambling transformation improves the security of the embedded watermark and increases the robustness of the proposed method. Two-dimensional Arnold scrambling transformation is defined as follows:

2.6 $$\left|\begin{array}{c} i^{\prime }\\ j^{\prime } \end{array}\right|=\left|\begin{array}{cc} 1 & 1\\ 1 & 2 \end{array}\right|\left|\begin{array}{c} i\\ j \end{array}\right|\text{ mod }N;\;i,j,i^{\prime },j^{\prime }=\{0,1,2,\ldots ,N-1\},$$

where *i*, *j* is the pixel coordinate of the original space; $i^{\prime }$, $j^{\prime }$is the pixel coordinate after iterative computation scrambling and *N* is the size of the watermark. To restore the original watermark, the corresponding inverse transformation formula can be defined as

2.7 $$\left|\begin{array}{c} i\\ j \end{array}\right|=\left(\left|\begin{array}{cc} 2 & -1\\ -1 & 1 \end{array}\right|\left|\begin{array}{c} i^{\prime }\\ j^{\prime } \end{array}\right|+\left|\begin{array}{c} N\\ N \end{array}\right|\right)\;\text{mod }N.$$

## 3. Proposed scheme

In this section, DCT-SVD based proposed watermarking scheme is elaborated in detail.

### 3.1. Watermark embedding procedure

**Figure 2.**
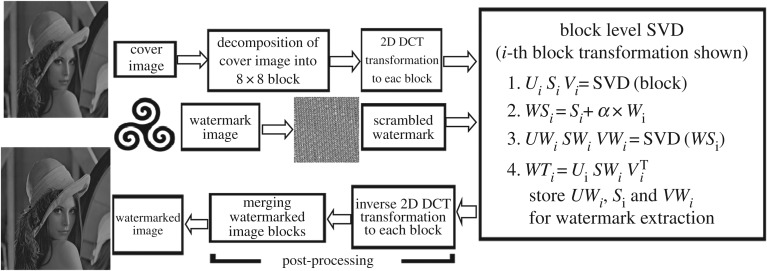
Embedding block diagram.

Assume *I* represents the cover image of size *M* × *N* and *W* represents the watermark image of size *M*1 × *N*1 (figure 2). To embed *W* into the *I*, proposed DCT-SVD based watermark embedding method steps are as follows:

*Step 1*: *Block processing of cover image*: The cover image *I* is divided into 8 × 8 non-overlapping blocks according to the following equation:

3.1 $$I(i,j)=\text{nob}\times I\text{\_}b_{p},$$

where 1 ≤ *i* ≤ *M*, 1 ≤ *j* ≤ *N*; nob = number of 8 × 8 non-overlapping cover image blocks = (*M* × *N*)/(8 × 8); and *I*_b_p_ is the divided 8 × 8 non-overlapping cover image block where 1 ≤ *p* ≤ nob.

*Step 2: Selection of 8* *×* *8 cover image block for watermark component embedding*: For security and authentication purposes, selection of watermark embedding position in cover image is one of the main concerns in the watermarking schemes. In this proposed scheme, one 8 × 8 non-overlapping cover image block is selected after four 8 × 8 non-overlapping cover image block and it starts from *I*_*b*_1_ (1st 8 × 8 non-overlapping cover image block). In other words, in each four 8 × 8 non-overlapping cover image blocks, the first block is selected for watermark module embedding purpose, i.e. *I*_*b*_1_ (1st 8 × 8 non-overlapping cover image block), *I*_*b*_5_ (5th 8 × 8 non-overlapping cover image block), *I*_*b*_9_ (9th 8 × 8 non-overlapping cover image block), … and so on till total 8 × 8 non-overlapping cover image block is traversed. So number of selected 8 × 8 non-overlapping cover image block for watermark component embedding is equal to nob/4 or 1/4th of total number of 8 × 8 non-overlapping cover image block. Keep these watermark embedding block values as the secret keys.

(For this proposed embedding algorithm, watermark size is considered as 1/4th of cover image. So, embedded blocks are selected one among four successive blocks. If watermark size is 1/2 of cover image, embedded blocks are selected one among two successive blocks. Similarly, if watermark size is 3/4th of cover image, embedded blocks are selected three among four successive blocks. In this way, if watermark size is equal to the cover image, all the cover image blocks are needed for watermark embedding purpose. So in this proposed method, watermark embedding capacity can be varied according to the size of watermark image.)

*Step 3*: *DCT transformation of selected 8* *×* *8 cover image blocks*: As discussed above, hybrid domain watermarking scheme provides better result than its single domain counterpart. So apply the DCT transformation to each selected 8 × 8 non-overlapping cover image block for watermark section embedding according to the following equation:

3.2 $$I\text{\_}b\;\text{dc}{\text{t}}_{p}=\text{dct( }I\_b_{p}\text{) },$$

where *I*_*b *dct*_p_* is the DCT transformed 8 × 8 cover image block where 1 ≤ *p* ≤ nob/4.

*Step 4*: *Encrypting and block processing the watermark*: At first, greyscale watermark *W* is encrypted to AW by Arnold scrambling technique. Then, scrambled watermark AW is partitioned into 8 × 8 non-overlapping blocks according to the following equation:

3.3 $$\text{AW}(i,j)=w_{\mathrm{nob}}\times W\text{\_}b_{p}(8,8),$$

where 1 ≤ *i* ≤ *M*1, 1 ≤ *j* ≤ *N*1, *w*_nob_ = number of 8 × 8 non-overlapping watermark blocks = (*M*1 × *N*1)/(8 × 8) and *W*_*b_p_* is the divided 8 × 8 non-overlapping watermark blocks, where 1 ≤ *p* ≤ *w*_nob_.

*Step 5*: *SVD transformation of DCT transformed cover image block*: Apply SVD to each DCT transformed 8 × 8 cover image blocks according to the following equation:

3.4 $$I\text{\_}b\;\text{dc}{\text{t}}_{p}=U_{p}\times S_{p}\times V_{p}^{T}.$$

*Step 6*: *Watermark embedding*: Embed the 8 × 8 non-overlapping scrambled watermark blocks into the SVs of DCT-SVD transformed 8 × 8 cover image blocks (*S_p_*) by multiplying with a scaling factor of *α* according to the following equation:

3.5 $$WS_{p}=S_{p}+\alpha \times W\text{\_}b_{p},$$

where 1 ≤ *p* ≤ *w*_nob_

*Step 7*: *SVD transformation of watermark embedded SVs*: Apply SVD to each watermark embedded 8 × 8 SVs block according to the following equation:

3.6 $$WS_{p}=UW_{p}\times SW_{p}\times VW_{p}^{T},$$

where 1 ≤ *p* ≤ *w*_nob_

*Step 8*: *Inverse SVD transformation of SVD transformed watermark embedded SVs*: Apply inverse SVD to the SVD transformed watermark embedded 8 × 8 SVs blocks according to the following equation:

3.7 $$U_{p}\times SW_{p}\times V_{p}^{T}=WT_{p},$$

where 1 ≤ *p* ≤ *w*_nob_

*Step 9*: *Inverse DCT transformation to each inverse SVD applied block*: Apply inverse DCT to each inverse SVD transformed 8 × 8 blocks according to the following equation:

3.8 $$I\text{\_}b\;\text{idc}{\text{t}}_{p}=\text{idct( }WT_{p}\text{)},$$

where 1 ≤ *p* ≤ *w*_nob_

*Step 10*: *Merging the divide image blocks*: Merge the divided 8 × 8 watermarked cover image blocks with other unchanged 8 × 8 cover image block (if unchanged cover image block exists) to construct final watermarked image. (In this proposed method, it can be easily proved that there will be no unchanged cover image block, if cover image block size is equal to the watermark image size.)

### 3.2. Watermark extracting procedure

**Figure 3.**
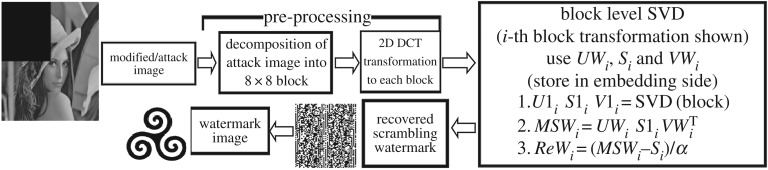
Extracting block diagram.

Assume *I*_attack represents the modified watermarked image of size *M* × *N* (figure 3). To recover watermark image of size *M*1 × *N*1 from modified watermarked image, proposed DCT-SVD based watermark extracting technique steps are as follows:

*Step 1*: *Block processing of modified watermarked image*: The modified watermarked image *I*_attack is divided into 8 × 8 non-overlapping blocks according to the following equation:

3.9 $$I\text{\_attack( }i,j\text{) }=\text{nob}\times I\text{\_attack}\;b_{p},$$

where 1 ≤ *i* ≤ *M*, 1 ≤ *j* ≤ *N*, nob = number of 8 × 8 non-overlapping modified watermarked image blocks = (*M* × *N*)/(8 × 8), and *I*_attack *b*_*p*_ is the divided 8 × 8 non-overlapping modified watermarked image block, where 1 ≤ *p* ≤ nob.

*Step 2*: *Selection of 8* *×* *8 modified watermarked image block for extracting watermark component using secret key*: In this proposed method, one 8 × 8 non-overlapping cover image block is selected after four 8 × 8 non-overlapping cover image blocks for watermark insertion. So, one 8 × 8 non-overlapping modified watermarked image block is selected after four 8 × 8 non-overlapping modified watermarked image blocks for watermark module extraction.

*Step 3*: *DCT transformation of selected 8 × 8 modified watermarked image blocks*: Apply the DCT transformation to each selected 8 × 8 non-overlapping modified watermarked image block for watermark section extracting according to the following equation:

3.10 $$I\text{\_attack}\;b\;\text{dc}{\text{t}}_{p}\text{ = dct (}I\text{\_attack}\;b_{p}\text{)},$$

where *I*_attack*b*dct*_p_* is the dct transformed 8 × 8 modified watermarked image block, where 1 ≤ *p* ≤ nob/4.

*Step 4*: *SVD transformation of DCT transformed modified watermarked image blocks*: Apply SVD to each DCT transformed 8 × 8 modified watermarked image blocks according to the following equation:

3.11 $$I\text{\_attack}\;b\;\text{dc}{\text{t}}_{p}=U1_{p}\times S1_{p}\times V1_{p}^{T},$$

where 1 ≤ *p* ≤ nob/4.

*Step 5*: *Inverse SVD transformation of DCT-SVD transformed modified watermarked image blocks*: Inverse SVD is applied to the 8 × 8 DCT-SVD transformed modified watermarked image blocks according to the following equation:

3.12 $$UW_{p}\times S1_{p}\times VW_{p}^{T}=\text{MS}{\text{W}}_{p},$$

where 1 ≤ *p* ≤ *w*_nob_

*Step 6*: *Encrypted watermark extracting*: Extract the 8 × 8 non-overlapping modified scrambled watermark blocks using the 8 × 8 modified watermarked image blocks (MSW*_p_*), SVs of DCT-SVD transformed 8 × 8 cover image blocks (*S_p_*) and value of α according to the following equation:

3.13 $$\text{Re}{\text{W}}_{p}=\frac{\left(\text{MS}{\text{W}}_{p}-S_{p}\right)}{\alpha },$$

where 1 ≤ *p* ≤ *w*_nob_

*Step 7*: *Recovering the watermark*: Merge the recovered 8 × 8 modified scrambled watermark blocks into one block to produce scrambled watermark. Watermark is recovered after applying Arnold scrambling in the following step.

## 4. Experimental result

To evaluate the performance of the proposed schemes, a number of experiments are performed in the Matlab platform on four standard greyscale benchmark images of size 512 × 512, namely Lena, Baboon, Barbara and Pepper images and a 256 × 256 logo image taken as a watermark image. The proposed DCT-SVD based watermarking schemes are tested with various experiments in terms of imperceptibility, robustness, security and capacity analysis.

### 4.1. Imperceptibility analysis

To calculate the imperceptibility/invisibility analysis, alteration of perceptual image quality (by the proposed watermarking method) should be determined. The peak signal-to-noise ratio (PSNR) is used to find perceptual similarity between a host image and a watermarked image. In an effective invisible watermarking algorithm (i) watermark should be imperceptible/invisible from human visual system (HVS) and should check with (ii) standard benchmark PSNR. PSNR values are presented in decibel (dB). For optimized imperceptibility, the minimum acceptable value of PSNR is 38 dB as suggested by Kutter & Petitcolas [25]. This convention is dubious because PSNR is not a meaningful constraint in the context of geometric distortions [26]. PSNR can be defined as follows:

4.1 $$\text{PSNR}=10\times \log_{10}\frac{{\text{max(}x(i,j)\text{)}}^{2}}{\text{MSE}},$$

where the mean square error (MSE) between host image *x* and the watermarked image $\bar{x}$ is defined as follows:

4.2 $$\text{MSE}=\frac{1}{M\times N}\sum_{i=1}^{M}\sum_{j=1}^{N}{\left(x_{ij}-{\bar{x}}_{ij}\right)}^{2},$$

where the notations *M* and *N* represent the width and height of an image, respectively; $x_{ij}$ is the pixel intensity value of coordinate (*i*, *j*) of the original image; and ${\bar{x}}_{ij}$ is the corresponding value of watermarked image. Basically, PSNR has been computed to compare the visual quality between cover image and watermarked image after embedding the watermark. Table 1 summarizes the experimental results for the proposed watermarking scheme in the context of PSNR value without any modification/attack of watermarked image.

**Table 1.** Imperceptibility and robustness measurement table for proposed scheme with PSNR, normalized correlation (NC) and bit error rate (BER) without attack.

image name	PSNR (in dB)	NC	BER
Lena	50.5281	0.9945	0.0085
Pepper	51.4004	0.9938	0.0094
Baboon	53.9286	0.9948	0.0081
Barbara	51.4541	0.9947	0.0078

### 4.2. Robustness analysis

The robustness indicates that the watermarked object should resist against some watermark removal intentional/unintentional attacks. To measure the robustness property of proposed method, bit error rate (BER) and normalized correlation (NC) value between the original watermark and extracted distorted watermark (without attack/after applying different types of attack) is compared. The range of NC value lies between −1 and +1. If watermarked image is almost similar to the original image, then this correlation value is approximately 1, while −1 correlation value indicates negative image like watermarked image. It becomes totally unacceptable or uncorrelated, if the NC value tends to 0. NC is considered to be acceptable if it is 0.75 [23] or above. The BER and NC can be calculated as follows:

4.3 $$\text{BER}=\frac{\text{Number of error bits}}{\text{Total bits transmitted}}=\frac{\text{Number of error bits per second}}{\text{Data rate per second}}$$

and

**Figure 4.**
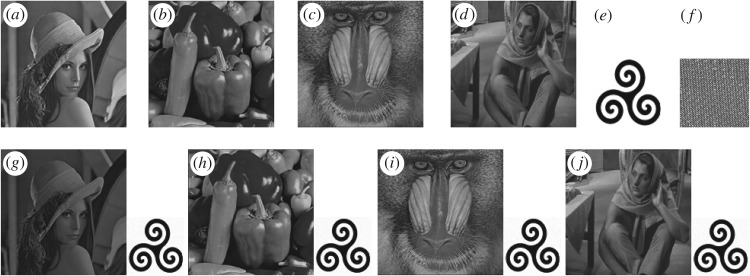
(*a*)–(*d*) Original test images, (*e*) the watermark of size, (*f*) the scrambled watermark, (*g*)–(*j*) all watermarked images and corresponding recovered watermarks without attack.

4.4 $$\text{NC}(w,\bar{w})=\frac{\sum_{i=1}^{M}\sum_{j=1}^{N}\left[w(i,j)-\mu_{w}]\times [\bar{w}(i,j)-\mu_{\bar{w}}\right]}{\sqrt{\sum_{i=1}^{M}\sum_{j=1}^{N}{\left[w(i,j)-\mu_{w}\right]}^{2}}\times \sqrt{\sum_{i=1}^{M}\sum_{j=1}^{N}{\text{[ }\bar{w}(i,j)-\mu_{\bar{w}}\text{] }}^{2}}},$$

where *M* and *N* represent the width and height of the watermark image, respectively; *w*(*i*,*j*) = the pixel intensity value at coordinate (*i*,*j*) of original watermark, $\bar{w}(i,j)$ = the pixel intensity value at coordinates (*i*,*j*) of extracting watermark, *µ*_*w*_ = mean of the original watermark, $\mu_{\bar{w}}$ = mean of the extracted watermark. BER and NC values of extracting watermark are calculated without any modification/attack of watermarked image. These values are presented in table 1. All the test images, watermark logo, encrypted watermark logo, watermarked images and recovered watermark logos from the corresponding watermarked images are shown side by side in figure 4.

### 4.3. Security analysis

As discussed in the introduction section, there are at least three serious drawbacks in SVD based algorithm: (i) *lower invisibility*, (ii) *false positive detection problem* and (iii) *diagonal line.* To overcome lower invisibility and diagonal line, SVs of cover image are modified to embed watermark instead of watermark SVs. Recently, scientists have proposed lots of ways to solve the false positive detection problem. In [20], Loukhaoukha *et al.* suggest to encrypt the watermark before inserting it into the cover object. In this proposed scheme, this encryption policy is employed to overcome the false positive detection issue. So, watermark logo is encrypted in this technique using Arnold scrambling before inserting into SVs of host image.

### 4.4. Capacity analysis

For this proposed embedding algorithm, watermark size is considered as 128 × 128. So, embedded blocks are selected one among four successive blocks. If watermark size is 256 × 256, embedded blocks are selected one among two successive blocks. Similarly, if watermark size is 384 × 384, embedded blocks are selected three among four successive blocks. In this way, if watermark size is 512 × 512, all the cover image blocks are needed for the watermark embedding purpose. So in this proposed method, watermark embedding capacity can be varied.

For performance evaluation and fair benchmarking, the proposed technique is verified against various attacks. In [26], Stirmark benchmark is represented as a watermarking scheme benchmark where different types of image watermarking scheme attacks are divided into categories such as signal enhancement, compression, scaling, cropping, shearing, rotation, linear transformations, other geometric transformations and random geometric distortions. These attacks are divided into three sections according to their properties as described in [25]. As a representative, Lena image figure has been shown under different types of attacks.

### 4.5. Enhancement technique attacks

**Figure 5.**
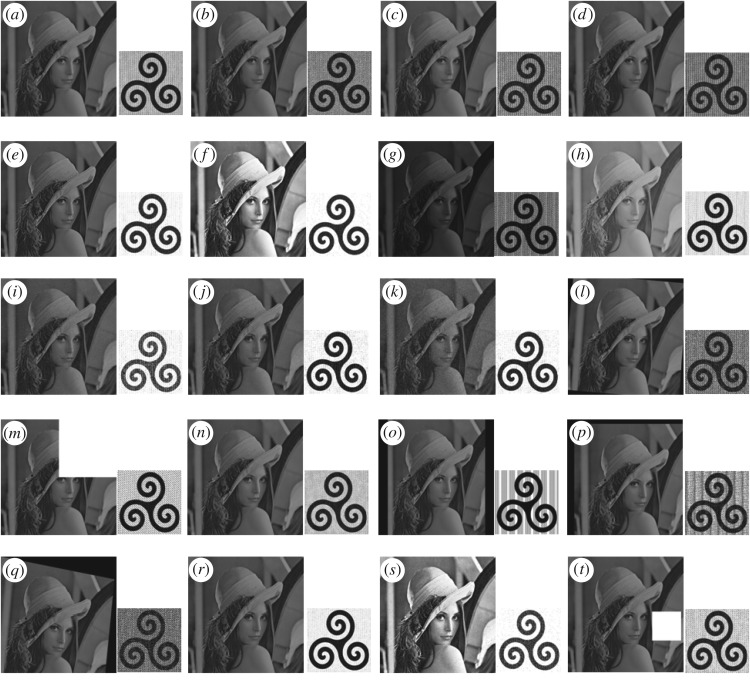
Result under different types of attack and recovered watermark images using (*a*) Gaussian low-pass filter (3,3), (*b*) median filter (3,3), (*c*) Wiener filter (3,3), (*d*) average filter (3,3), (*e*) image sharpening, (*f*) histogram equalization, (*g*) gamma attack (*γ* = 2), (*h*) gamma attack (*γ* = 0.5), (*i*) salt and pepper noise (density = 0.01), (*j*) speckle noise (var = 0.01), (*k*) Gaussian noise (*M* = 0, var = 0.01), (*l*) rotation clockwise 5°, (*m*) cropping (256 × 256 by white), (*n*) scaling (zoomout = 2, zoomin = 0.5), (*o*) cut (40 columns), (*p*) translation (30,20), (*q*) image shearing (0.1, −0.2), (*r*) JPEG compression, *Q* = 50, (*s*) histogram equalization + salt and pepper noise (density = 0.01) and (*t*) JPEG compression *Q* = 50 + cropping (128 × 128 by white).

(i) Low-pass filtering: Low-pass filtering operation is used to remove high frequency noise from a signal. To prove the robustness of the proposed technique against low-pass filter, Gaussian low-pass filtering attack with six different size parameters (2 × 2, 3 × 3, 5 × 5, 7 × 7, 9 × 9, 11 × 11) are applied on selected standard test images. With the increment of filter order size, high frequency components of watermarked image are attenuated by the low-pass filtering operation. Modified watermarked image under Gaussian low-pass filtering attack (3 × 3) and its corresponding recovered watermark logo are presented in figure 5*a*. BER and NC values of recovered watermark, from table 2, conclude that recovered watermark data of this proposed method survive the Gaussian low-pass manipulation filtering attack. Data from table 2 establish the consistency of recovered watermark data in the Gaussian low-pass filtering attack.
(ii) Median filtering: In common image enhancement application, a median filter is not often used to achieve blurring rather than it is used in the noise reduction process. Basically, median filter modifies the centre pixel value of the window with the middle value of the sorted pixel values. The proposed scheme is examined against median filtering attacks with different window sizes. Recovered watermark logo and its corresponding median filtering (3 × 3) modified image are shown in figure 5*b*. BER and NC values of recovered watermark prove the robustness and steadiness of this scheme against median filtering attack. BER and NC values of recovered watermark in table 3 provide enough evidence to demonstrate the steadiness of the method.
(iii) Wiener filtering: To simulate the effects of two-dimensional linear predictive image coding, Wiener filter is applied. With the improvement of prediction filter order size, the model becomes closer to the watermarked image block. So, estimation-based attack becomes easier, if the attacker has some basic knowledge about the watermark's embedding process. Image denoising is one of the malicious estimation based attacks. This technique is tested against Wiener filtering attack under different size parameters. Wiener filtering modified image and corresponding logo is depicted in figure 5*c*. Analyses of NC and BER values of table 4 exhibit the consistency of the recovered watermark data and confirm the reliability of the proposed scheme.
(iv) Average filtering: Average filtering is one of the well known image enhancement technique attacks and one of the denoising attacks. An average filter is applied to replace each sample of the watermarked image with the average value from the set of *W* × *W* neighbouring pixels or *W* window size. This scheme is verified against average filtering attack, as shown in figure 5*d*. NC and BER values of recovered watermark data demonstrate the inverse proportionality and proportionality with the window size. This is provided in table 5.
(v) Gamma correction: Gamma correction is one of the popular image enhancement techniques to adjust poor image display quality. Also, sometimes intentionally or unintentionally, image is enhanced by power law transformation or gamma correction method. Figure 5*g*,*h* shows watermarked image after applying gamma correction with two different gamma values. Consistency of NC and BER values of recovered watermark data in shown in table 6.
(vi) Image sharpening: Sharpening attack is used to detect high-frequency noise introduced by unauthorized users. This proposed method is tested against sharpening operation as shown in figure 5*e*. NC and BER values of recovered watermark data (under image sharpening image enhancement attack) are given in table 7.
(vii) Histogram modification: To compensate the clarity and brightness of an image, sometimes histogram of the image is modified. The histogram modification, enhancement technique is common to improve the visual quality of any image. The proposed technique is tested against histogram modification operation as shown in figure 5*f*. NC and BER values of recovered watermark data are given in table 7.

**Table 2.** NC and BER of the extracted watermark under Gaussian low-pass filter attack to Lena, Pepper, Baboon and Barbara image.

	Lena		Pepper		Baboon		Barbara	
attack	NC	BER	NC	BER	NC	BER	NC	BER
(A) Gaussian low-pass filter (2,2)	0.8012	0.2182	0.8062	0.2092	0.8424	0.1756	0.7913	0.2144
(B) Gaussian low-pass filter (3,3)	0.8269	0.1938	0.8172	0.2035	0.8498	0.1789	0.8183	0.2079
(C) Gaussian low-pass filter (5,5)	0.8291	0.1912	0.8169	0.2047	0.8493	0.1902	0.8183	0.2079
(D) Gaussian low-pass filter (7,7)	0.8291	0.1912	0.8169	0.2047	0.8493	0.1902	0.8183	0.2079
(E) Gaussian low-pass filter (9,9)	0.8291	0.1912	0.8169	0.2047	0.8493	0.1902	0.8183	0.2079
(F) Gaussian low-pass filter (11,11)	0.8291	0.1912	0.8169	0.2047	0.8493	0.1902	0.8183	0.2079

**Table 3.** NC and BER of the extracted watermark under median filter attack to Lena, Pepper, Baboon and Barbara image.

	Lena		Pepper		Baboon		Barbara	
attack	NC	BER	NC	BER	NC	BER	NC	BER
(A) median filter (2,2)	0.8019	0.2013	0.8054	0.2032	0.8483	0.1718	0.8029	0.2042
(B) median filter (3,3)	0.7825	0.2392	0.7840	0.2295	0.7933	0.2061	0.7832	0.2235
(C) median filter (5,5)	0.7741	0.2665	0.7791	0.2470	0.7818	0.2174	0.7747	0.2355
(D) median filter (7,7)	0.7660	0.2645	0.7882	0.2364	0.7541	0.2592	0.7660	0.2510
(E) median filter (9,9)	0.7606	0.2628	0.7865	0.2237	0.7480	0.2763	0.7620	0.2566
(F) median filter (11,11)	0.7607	0.2662	0.7520	0.2625	0.7446	0.2785	0.7641	0.2637

**Table 4.** NC and BER of the extracted watermark under Wiener filter attack to Lena, Pepper, Baboon and Barbara image.

	Lena		Pepper		Baboon		Barbara	
attack	NC	BER	NC	BER	NC	BER	NC	BER
(A) Wiener filter (2,2)	0.8281	0.1989	0.8265	0.2742	0.8714	0.1445	0.8351	0.1719
(B) Wiener filter (3,3)	0.7892	0.2366	0.8030	0.2021	0.8140	0.2072	0.7881	0.2259
(C) Wiener filter (5,5)	0.7803	0.2421	0.8012	0.2098	0.7682	0.2513	0.7664	0.2474
(D) Wiener filter (7,7)	0.7758	0.2389	0.7926	0.2180	0.7609	0.2574	0.7684	0.2432
(E) Wiener filter (9,9)	0.7608	0.2498	0.7879	0.2376	0.7537	0.2684	0.7544	0.2627
(F) Wiener filter (11,11)	0.7527	0.2559	0.7684	0.2513	0.7509	0.2690	0.7479	0.2682

**Table 5.** NC and BER of the extracted watermark under average filter attack to Lena, Pepper, Baboon and Barbara Image.

	Lena		Pepper		Baboon		Barbara	
attack	NC	BER	NC	BER	NC	BER	NC	BER
(A) average filter (2,2)	0.8012	0.2082	0.8062	0.2096	0.8424	0.1756	0.7913	0.2144
(B) average filter (3,3)	0.7617	0.2556	0.7772	0.2334	0.7854	0.2124	0.7772	0.2413
(C) average filter (5,5)	0.7581	0.2573	0.7771	0.2339	0.7692	0.2397	0.7647	0.2406
(D) average filter (7,7)	0.7547	0.2638	0.7649	0.2446	0.7641	0.2445	0.7530	0.2688
(E) average filter (9,9)	0.7494	0.2622	0.7522	0.2653	0.7542	0.2672	0.7446	0.2637
(F) average filter (11,11)	0.7431	0.2761	0.7428	0.2704	0.7510	0.2663	0.7384	0.2755

**Table 6.** NC and BER of the extracted watermark under gamma correction attack to Lena, Pepper, Baboon and Barbara image.

	Lena		Pepper		Baboon		Barbara	
attack	NC	BER	NC	BER	NC	BER	NC	BER
(A) gamma attack (*γ* = 3)	0.8931	0.2207	0.8830	0.1325	0.8781	0.1403	0.8561	0.1407
(B) gamma attack (*γ* = 2)	0.9107	0.1092	0.9159	0.1003	0.9002	0.1032	0.9255	0.0937
(C) gamma attack (*γ* = 0.75)	0.9669	0.0523	0.9649	0.0558	0.9316	0.0810	0.9539	0.0623
(D) gamma attack (*γ* = 0.5)	0.9523	0.0667	0.9265	0.0971	0.8730	0.1353	0.8810	0.1369
(E) gamma attack (*γ* = 0.25)	0.8754	0.1498	0.8797	0.1249	0.8516	0.1433	0.7879	0.1915

**Table 7.** NC and BER of the extracted watermark under different types of attack to Lena, Pepper, Baboon and Barbara image.

	Lena		Pepper		Baboon		Barbara	
attack	NC	BER	NC	BER	NC	BER	NC	BER
(A) image sharpening	0.9529	0.0615	0.9427	0.0723	0.9315	0.0736	0.9428	0.0707
(B) histogram equalization	0.9530	0.0668	0.9806	0.0344	0.9613	0.0536	0.9775	0.0373

### 4.6. Noise addition attack

(viii) Noise addition: Most addressed non-geometrical attack in signal processing is the addition of additive noise and uncorrelated multiplicative noise. The proposed scheme is tested against salt and peppers, speckle and Gaussian noise.

Salt and pepper noise is caused by pixel's error at the time of data transmission. In salt and pepper noise, corrupted pixel values are either set to zero or maximum value or single bits flipped over. This pixel's value modification gives the image salt and pepper-like appearance where noise density is calculated by the alteration of percentage of pixels. This method is tested against salt and pepper noise with six different noise densities. Salt and pepper noise modified watermarked image with noise density 0.01 is shown in figure 5*i*. In salt and pepper noise, inverse proportionality of NC and proportionality of BER of recovered watermark with noise density is given in table 8.

**Table 8.** NC and BER of the extracted watermark under salt and pepper noise attack to Lena, Pepper, Baboon and Barbara image.

	Lena		Pepper		Baboon		Barbara	
attack	NC	BER	NC	BER	NC	BER	NC	BER
(A) salt and pepper noise (density = 0.5)	0.8384	0.1748	0.8357	0.1851	0.8294	0.1939	0.8486	0.1782
(B) salt and pepper noise (density = 0.3)	0.8599	0.1641	0.8497	0.1679	0.8526	0.1637	0.8692	0.1460
(C) salt and pepper noise (density = 0.1)	0.8793	0.1280	0.8604	0.1460	0.8984	0.1127	0.8981	0.1200
(D) salt and pepper noise (density = 0.05)	0.9009	0.1065	0.8871	0.1315	0.9074	0.1031	0.9048	0.1031
(E) salt and pepper noise (density = 0.03)	0.9137	0.0932	0.9096	0.1034	0.9114	0.1217	0.9211	0.0901
(F) salt and pepper noise (density = 0.01)	0.9386	0.0739	0.9102	0.0875	0.9537	0.0746	0.9362	0.0873

Speckle noise is one of the multiplicative noises where speckle exists inherently as a granular noise. The variance of a single pixel is equal to the variance of the local area that is centred on that pixel. This method is tested against speckle noise under six different noise variances. Manipulated watermarked image by speckle noise with variance = 0.01 is portrayed in figure 5*j*. NC values of recovered watermark exhibit inverse proportionality with speckle image noise variance where BER shows proportionality characteristic. This is tabulated in table 9.

**Table 9.** NC and BER of the extracted watermark under speckle noise attack to Lena, Pepper, Baboon and Barbara image.

	Lena		Pepper		Baboon		Barbara	
attack	NC	BER	NC	BER	NC	BER	NC	BER
(A) speckle noise (var = 0.5)	0.9004	0.1063	0.9199	0.1017	0.8978	0.1247	0.9397	0.0696
(B) speckle noise (var = 0.3)	0.9233	0.0950	0.9325	0.0790	0.9371	0.0864	0.9598	0.0678
(C) speckle noise (var = 0.1)	0.9453	0.0742	0.9435	0.0712	0.9403	0.0786	0.9610	0.0667
(D) speckle noise (var = 0.05)	0.9703	0.0597	0.9590	0.0634	0.9476	0.0718	0.9643	0.0597
(E) speckle noise (var = 0.03)	0.9750	0.0582	0.9635	0.0540	0.9545	0.0634	0.9667	0.0508
(F) speckle noise (var = 0.01)	0.9859	0.0464	0.9743	0.0485	0.9705	0.0498	0.9783	0.0453

Gaussian noise is one of the commonly used statistical noise processing operations. The amount of noise is varied by its variance with zero mean. For robustness clarification, this statistical noise is added to the watermarked images of this method. Additions of Gaussian noise under different variances are depicted in table 10. Watermarked image with additive Gaussian noise (variance = 0.01, mean = 0) is described in figure 5*k*.

**Table 10.** NC and BER of the extracted watermark under Gaussian image noise attack to Lena, Pepper, Baboon and Barbara image.

	Lena		Pepper		Baboon		Barbara	
attack	NC	BER	NC	BER	NC	BER	NC	BER
(A) Gaussian noise (*M* = 0, var = 0.5)	0.9018	0.0893	0.8929	0.1260	0.9084	0.1099	0.9163	0.0983
(B) Gaussian noise (*M* = 0, var = 0.3)	0.9123	0.0750	0.9024	0.1079	0.9185	0.0924	0.9246	0.0832
(C) Gaussian noise (*M* = 0, var = 0.1)	0.9252	0.0725	0.9159	0.0948	0.9298	0.0842	0.9395	0.0797
(D) Gaussian noise (*M* = 0, var = 0.05)	0.9362	0.0713	0.9276	0.0727	0.9384	0.0792	0.9491	0.0691
(E) Gaussian noise (*M* = 0, var = 0.03)	0.9490	0.0607	0.9396	0.0671	0.9407	0.0714	0.9512	0.0677
(F) Gaussian noise (*M* = 0, var = 0.01)	0.9557	0.0596	0.9451	0.0539	0.9443	0.0608	0.9577	0.0569

### 4.7. Geometric transformation attacks

(ix) Rotation: The commercial value of an image and its small rotated version is not differing too much. But watermark data can be affected by the little bit of rotation. To verify robustness against rotation geometric attack, the proposed technique is tested against rotation attack with different angles in clockwise and anticlockwise directions. BER and NC values of recovered watermark image are demonstrated in table 11. Figure of rotation attack under 5° is presented in figure 5*l*. Recovered watermark image, after rotation attack, is also given in the same figure. This image is not obscure and distinctively represents the watermark logo.
(x) Cropping: To capture the required part of an object, cropping/focusing is done on the necessary part. The rest of an object or image is neglected. Cropping operation is also used to change aspect ratio of an image or to improve image framing. Robustness of the proposed technique is tested against cropping operation by changing the cropping window size. Watermarked images of the proposed scheme are tested against cropping geometric attack under different cropping window sizes. For this proposed method, 256 × 256, 128 × 128 and 64 × 64 cropping window size is used. This cropping window may be black or white alternatively. One cropping attack image where 256 × 256 pixels are replaced by white window is shown in figure 5*m*. Recovered watermark images NC and BER values provide enough evidence of the stability of the proposed method. Recovered watermark images NC and BER values are presented in table 12.
(xi) Scaling: Image may be resized intentionally or unintentionally in uniform/non-uniform manner. For example, scaling of the image is a normal scenario for scanning of a hard copy image. So, the proposed scheme is tested against scaling operation to prove the robustness. At first, the image is resized by multiplying by a scaling factor. Then again the image is scaled back to its original size. As a representative, one watermarked image and corresponding recovered logo under this scaling geometric attack are represented in figure 5*n*. NC and BER values of recovered watermark images are given in table 13.
(xii) Deletion of lines or columns: Very frequently some part of the image is removed or deleted intentionally or unintentionally. This is one of the geometric transformation attacks that have been widely used against some simple copyright protection watermarking systems. Sometimes, this deletion of lines or columns has the same effect as image scaling. The proposed technique is verified against this type of attack for better robustness. In this experiment, some lines (rows) or columns of watermarked images are deleted. For row deletion, some upper rows and lower rows, where row numbers are the same, are deleted. For column deletion, the same numbers of columns from left and right side of the watermarked images are deleted. One 40 column deleted watermarked image is shown in figure 5*o*. To prove the consistency of the proposed scheme, NC and BER values of recovered watermark images under different row and column sizes are shown in table 14.
(xiii) Translation: Sometimes, deletion of part of an image and geometric transformation of the remaining part of the image is done in such a way that it looks like the image is translated into a new coordinate. To provide better robustness against translation attack, the proposed technique is verified against it by varying translation coordinate. In figure 5*p*, one translation attack image with translation coordinate (30,20) is presented and a corresponding recovered watermark is shown. To explicitly confirm the robustness of the proposed scheme, NC and BER values of recovered watermark image are shown in table 15.
(xiv) Shearing: To confirm the robustness against shear operation, the proposed technique is tested against image shearing with different parameters. Actually, shearing of an image means distortion of the image in the *x*-direction or *y*-direction or in both directions. Here one watermarked image is sheared in both directions with different parameter values, as shown in figure 5*q*. Recovered watermark images BER and NC values are tabulated in table 16 to prove robustness consistency under shearing operation.

**Table 11.** NC and BER of the extracted watermark under rotation attack to Lena, Pepper, Baboon and Barbara image.

	Lena		Pepper		Baboon		Barbara	
attack	NC	BER	NC	BER	NC	BER	NC	BER
(A) rotation (clockwise 5°)	0.8246	0.2010	0.8659	0.1522	0.8448	0.1775	0.8331	0.1671
(B) rotation (anticlockwise 5°**)**	0.8133	0.2139	0.8601	0.1534	0.8432	0.1678	0.8395	0.1719
(C) rotation (clockwise 25°**)**	0.7671	0.2516	0.8055	0.2065	0.8042	0.2063	0.7876	0.2380
(D) rotation (anticlockwise 25°**)**	0.7734	0.2431	0.8074	0.2049	0.8028	0.2091	0.7756	0.2408
(E) rotation (clockwise 45°**)**	0.7541	0.2634	0.7856	0.2382	0.7726	0.2451	0.7452	0.2708
(F) rotation (anticlockwise 45°**)**	0.7605	0.2401	0.7848	0.2415	0.7625	0.2541	0.7501	0.2713

**Table 12.** NC and BER of the extracted watermark under cropping attack to Lena, Pepper, Baboon and Barbara image.

	Lena		Pepper		Baboon		Barbara	
attack	NC	BER	NC	BER	NC	BER	NC	BER
(A) cropping (256 × 256 by black)	0.9845	0.0327	0.9838	0.0446	0.9748	0.0420	0.9647	0.0452
(B) cropping (256 × 256 by white)	0.9898	0.0219	0.9889	0.0344	0.9782	0.0368	0.9680	0.0307
(C) cropping (128 × 128 by black)	0.9925	0.0125	0.9902	0.0204	0.9840	0.0357	0.9834	0.0221
(D) cropping (128 × 128 by white)	0.9932	0.0119	0.9942	0.0107	0.9853	0.0340	0.9846	0.0198
(E) cropping (64 × 64 by black)	0.9944	0.0090	0.9936	0.0097	0.9918	0.0181	0.9905	0.0149
(F) cropping (64 × 64 by white)	0.9947	0.0086	0.9939	0.0105	0.9920	0.0145	0.9927	0.0096

**Table 13.** NC and BER of the extracted watermark under scaling attack to Lena, Pepper, Baboon and Barbara image.

	Lena		Pepper		Baboon		Barbara	
attack	NC	BER	NC	BER	NC	BER	NC	BER
(A) scaling (zoomout = 0.5, zoomin = 2)	0.7926	0.2281	0.8029	0.2177	0.8282	0.1945	0.7891	0.2307
(B) scaling (zoomout = 0.25, zoomin = 4)	0.7832	0.2665	0.7968	0.2207	0.8032	0.2009	0.7996	0.2203
(C) scaling (zoomout = 0.125, zoomin = 8)	0.7761	0.2757	0.8253	0.1974	0.8504	0.1742	0.8409	0.1701
(D) scaling (zoomout = 2, zoomin = 0.5)	0.8644	0.1577	0.8485	0.1784	0.8976	0.1365	0.8581	0.1638
(E) scaling (zoomout = 4, zoomin = 0.25)	0.8665	0.1456	0.8496	0.1679	0.8987	0.1252	0.8599	0.1627
(F) scaling (zoomout = 8, zoomin = 0.125)	0.8672	0.1542	0.8507	0.1552	0.8986	0.1147	0.8601	0.1562

**Table 14.** NC and BER of the extracted watermark under deletion of lines or columns attack to Lena, Pepper, Baboon and Barbara image.

	Lena		Pepper		Baboon		Barbara	
attack	NC	BER	NC	BER	NC	BER	NC	BER
(A) cut (20 rows)	0.9890	0.0340	0.9889	0.0483	0.9914	0.0148	0.9894	0.0395
(B) cut (20 columns)	0.9905	0.0194	0.9939	0.0140	0.9948	0.0093	0.9947	0.0148
(C) cut (30 rows)	0.9807	0.0361	0.9803	0.0302	0.9861	0.0371	0.9816	0.0314
(D) cut (30 columns)	0.9882	0.0262	0.9876	0.0323	0.9883	0.0251	0.9910	0.0131
(E) cut (40 rows)	0.9604	0.0551	0.9725	0.0394	0.9756	0.0381	0.9637	0.0507
(F) cut (40 columns)	0.9642	0.0435	0.9738	0.0376	0.9618	0.0445	0.9648	0.0495

**Table 15.** NC and BER of the extracted watermark under translation attack to Lena, Pepper, Baboon and Barbara image.

	Lena		Pepper		Baboon		Barbara	
attack	NC	BER	NC	BER	NC	BER	NC	BER
(A) image translation (10,10)	0.8192	0.2013	0.8604	0.1679	0.8495	0.1808	0.8692	0.1679
(B) image translation (10,20)	0.8064	0.2124	0.8573	0.1776	0.8353	0.1948	0.8116	0.2065
(C) image translation (20,10)	0.7946	0.2197	0.8508	0.1818	0.8333	0.1989	0.8080	0.2178
(D) image translation (20,20)	0.7972	0.2294	0.8206	0.1940	0.7953	0.2286	0.7630	0.2558
(E) image translation (20,30)	0.7821	0.2384	0.7810	0.2333	0.7687	0.2565	0.7544	0.2621
(F) image translation (30,20)	0.7703	0.2554	0.7782	0.2467	0.7623	0.2399	0.7370	0.2969

**Table 16.** NC and BER of the extracted watermark under shearing attack to Lena, Pepper, Baboon and Barbara image.

	Lena		Pepper		Baboon		Barbara	
attack	NC	BER	NC	BER	NC	BER	NC	BER
(A) image shearing (0.1,0.1)	0.8556	0.1704	0.7945	0.2203	0.7947	0.2249	0.8262	0.1921
(B) image shearing (−0.1,0.1)	0.8299	0.1906	0.7686	0.2513	0.7897	0.2409	0.7927	0.2361
(C) image shearing (0.1, −0.1)	0.8217	0.2021	0.7582	0.2692	0.7806	0.2433	0.7883	0.2439
(D) image shearing (−0.1, −0.1)	0.8187	0.1971	0.7466	0.2730	0.7471	0.2827	0.7774	0.2512
(E) image shearing (−0.1,0.2)	0.7864	0.2310	0.7299	0.2899	0.7257	0.3275	0.7364	0.2966
(F) image shearing (0.1, −0.2)	0.7912	0.2411	0.7392	0.2764	0.7231	0.3156	0.7367	0.2881

### 4.8. Compression attack

(xv) JPEG compression: JPEG compression is one of the most widely used common manipulation/compression attacks. Any effective watermark should be rigid to some degree of compression. The proposed scheme is checked against JPEG compression by changing quality factor. One compression attack image (quality factor = 50) along with recovered logo is given in figure 5*r*. Recovered watermark logos NC and BER values from the compressed watermarked image under different quality factor are given in table 17. Results in the table provide enough data to confirm the improvement of recovered watermark quality with the increment of JPEG compression quality factor.

**Table 17.** NC and BER of the extracted watermark under JPEG attack to Lena, Pepper, Baboon and Barbara image.

	Lena		Pepper		Baboon		Barbara	
attack	NC	BER	NC	BER	NC	BER	NC	BER
(A) JPEG compression *Q* = 20	0.9019	0.1163	0.8897	0.1316	0.9396	0.0805	0.9071	0.1126
(B) JPEG compression *Q* = 25	0.9117	0.1024	0.8956	0.1253	0.9468	0.0723	0.9091	0.1020
(C) JPEG compression *Q* = 30	0.9263	0.0984	0.9018	0.1182	0.9559	0.0692	0.9327	0.0843
(D) JPEG compression *Q* = 40	0.9381	0.0853	0.9084	0.1005	0.9597	0.0676	0.9434	0.0579
(E) JPEG compression *Q* = 50	0.9500	0.0524	0.9162	0.0935	0.9644	0.0509	0.9479	0.0541
(F) JPEG compression *Q* = 70	0.9635	0.0450	0.9320	0.0756	0.9677	0.0495	0.9664	0.0448

### 4.9. Combinational attack

For robustness test, some combination of above discussed attacks is applied to all the standard test images. There can be lots of combinational attacks, but some of the different combinational attacks are presented as follows:

(xvi) Combinational attack: For robustness clarity, combination of one enhancement technique attack (histogram equalization) and one geometric attack (cropping (128 × 128 by white)); combination of one enhancement technique attack (histogram equalization) and one noise addition attack (salt and pepper noise (density = 0.01)); combination of one enhancement technique attack (histogram equalization) and one compression attack (JPEG compression *Q* = 50); combination of one noise addition attack (salt and pepper noise (density = 0.01)) and one geometric attack (cropping (128 × 128 by white)); combination of one noise addition attack (salt and pepper noise (density = 0.01)) and one compression attack (JPEG compression *Q* = 50); combination of one compression attack (JPEG compression *Q* = 50) and one geometric attack (cropping (128 × 128 by white)) are applied on watermarked images of the proposed scheme (as given in table 18). For combinational attack, two of the attack images of this combinational attack are portrayed in figure 5*s*,*t*. Recovered watermarks for these attacks are also given in the same figure (figure 5*s*,*t*).

**Table 18.** NC and BER of the extracted watermark under different combinational attacks to Lena, Pepper, Baboon and Barbara image.

	Lena		Pepper		Baboon		Barbara	
combinational attack	NC	BER	NC	BER	NC	BER	NC	BER
(A) histogram equalization + salt and pepper noise (density = 0.01)	0.8781	0.1413	0.8687	0.1580	0.8558	0.1629	0.8923	0.1208
(B) histogram equalization + cropping (128 × 128 by white)	0.9215	0.1090	0.9188	0.1036	0.9089	0.1037	0.9276	0.0903
(C) histogram equalization + JPEG compression *Q* = 50	0.9335	0.0889	0.8939	0.1204	0.9269	0.1061	0.9050	0.1289
(D) salt and pepper noise (density = 0.01) + cropping (128 × 128 by white)	0.8757	0.1391	0.8529	0.1716	0.8540	0.1655	0.8624	0.1597
(E) salt and pepper noise (density = 0.01) + JPEG compression *Q* = 50	0.8865	0.1475	0.8426	0.1889	0.8929	0.1242	0.8943	0.1263
(F) JPEG compression *Q* = 50 + cropping (128 × 128 by white)	0.8998	0.1142	0.9076	0.1215	0.9173	0.0916	0.9238	0.1052

### 4.10. Comparative analysis

**Figure 6.**
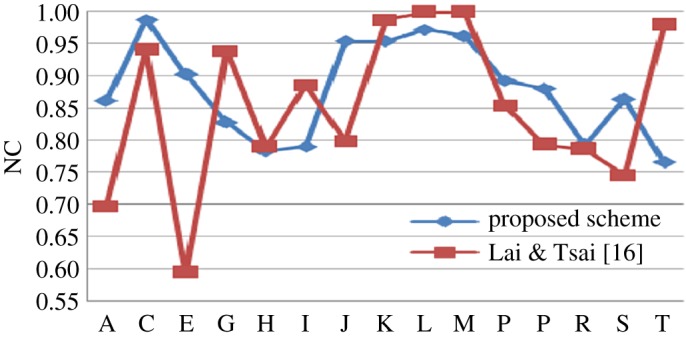
Comparison of proposed scheme with Lai & Tsai [16].

**Figure 7.**
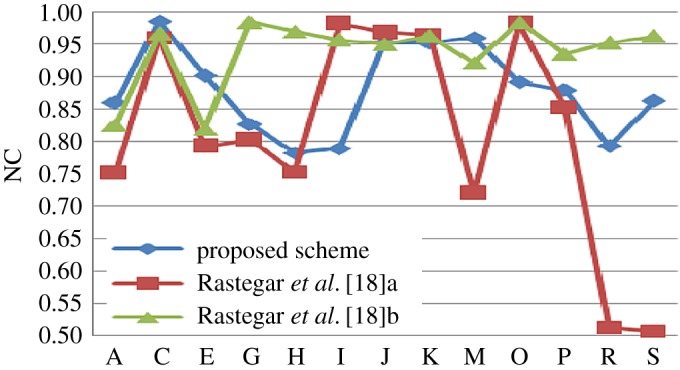
Comparison of proposed scheme with Rastegar *et al.* [18].

**Figure 8.**
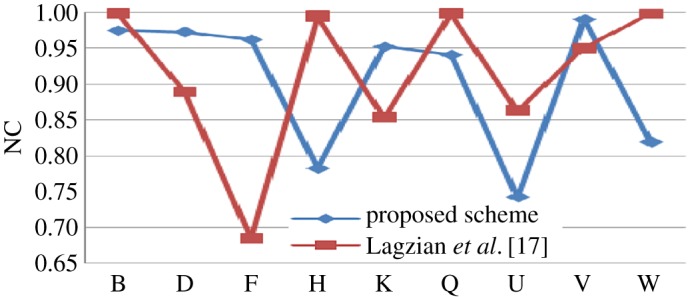
Comparison of proposed scheme with Lagzian *et al.* [17].

**Figure 9.**
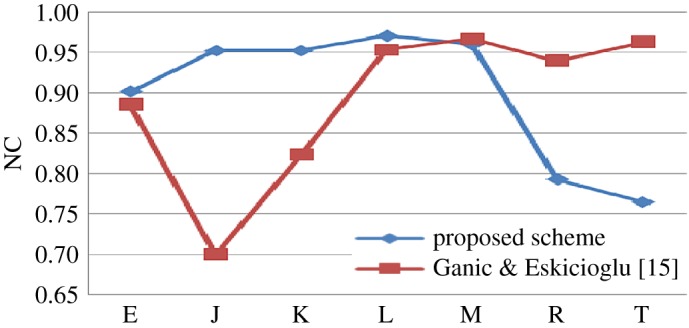
Comparison of proposed scheme with Ganic & Eskicioglu [15].

**Figure 10.**
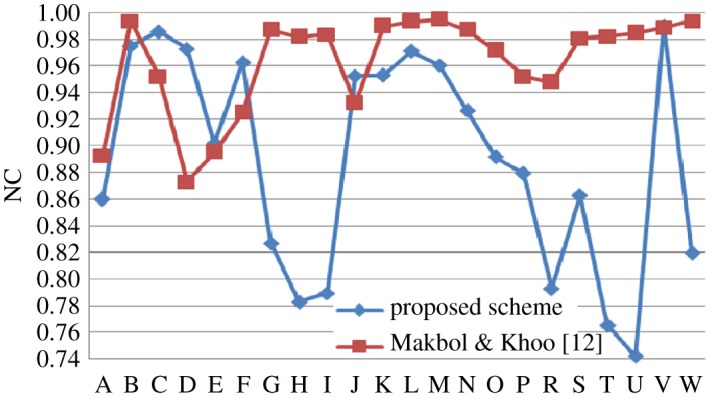
Comparison of proposed scheme with Makbol & Khoo [12].

The proposed scheme is compared with some existing algorithm in tabular format with some predefined attacks. Then individual comparison is done with some existing methods as graphical plot (figures 6–10). Better results are shown as bold data in table 19.

**Table 19.** Comparative analysis of proposed scheme with some existing scheme in terms of various attack (‘—’ represent this experiment is not done in this technique; better results are in bold).

attack	Proposed scheme	Lai & Tsai [16]	Rastegar *et al*. [18]a	Rastegar *et al*. [18]b	Lagzian *et al*. [17]	Ganic & Eskicioglu [15]	Makbol & Khoo [12]
(A) salt and pepper noise (density 0.3)	0.8599	0.6967	0.7515	0.8258	—	—	**0.8926**
(B) salt and pepper noise (density 0.001)	0.9753	—	—	—	**0.9985**	—	0.9940
(C) speckle noise (var = 0.01)	**0.9859**	0.9393	0.9609	0.9667	—	—	0.952
(D) speckle noise (var = 0.04)	**0.9729**	—	—	—	0.8896	—	0.8730
(E) Gaussian noise (*M* = 0, var = 0.5)	**0.9018**	0.5949	0.7926	0.82	—	0.8850	0.8953
(F) Gaussian noise (*M* = 0, var = 0.005)	**0.9627**	—	—	—	0.6838	—	0.9250
(G) Gaussian filtering (3 × 3)	0.8269	0.9374	0.8023	0.9843	—	—	**0.987**
(H) median filtering (3 × 3)	0.7825	0.7886	0.7534	0.9706	**0.9942**	—	0.982
(I) Wiener filtering (3 × 3)	0.7892	0.8845	0.9824	0.9569	—	—	**0.984**
(J) sharpening	**0.9529**	0.7984	0.9687	0.9511	—	0.6990	0.932
(K) histogram equalization	0.9530	0.9862	0.9648	0.9628	0.8530	0.8230	**0.990**
(L) gamma correction (0.7)	0.9712	0.9982	—	—	—	0.9540	**0.9935**
(M) gamma correction (0.8)	0.9608	**0.9980**	0.7203	0.9217	—	0.9675	0.9950
(N) JPEG compression *Q* = 30	0.9263	—	—	—	—	—	**0.987**
(O) JPEG compression *Q* = 10	0.8915	0.8532	0.9824	**0.9843**	—	—	0.972
(P) JPEG compression *Q* = 5	0.8794	0.7926	0.8532	0.9354	—	—	**0.952**
(Q) JPEG compression 50%	0.9414	—	—	—	**0.9983**	—	—
(R) scaling (zoomout = 0.5, zoomin = 2)	0.7926	0.7847	0.5127	0.953	—	0.9400	**0.948**
(S) rotation (angle = 2°)	0.8627	0.7456	0.5068	0.9628	—	—	**0.981**
(T) rotation (angle = −20°)	0.7651	0.9780	—	—	—	0.9630	**0.9823**
(U) rotation (angle = −50°)	0.7419	—	—	—	0.8630	—	**0.9850**
(V) cut 20	**0.9905**	—	—	—	0.9512	—	0.989
(W) shift 2%	0.8192	—	—	—	**0.9989**	—	0.9940

## 5. Conclusion

In this paper, a DCT-SVD based robust hybrid image watermarking scheme using Arnold scrambling has been proposed. Special attention is given to security issues such as (i) *lower invisibility*, (ii) *false positive detection problem* and (iii) *diagonal line* problem of SVD-based watermarking algorithms. The proposed scheme is tested against common image manipulation attacks, geometric transformation attacks and compression attacks to confirm the imperceptibility and robustness property effectively. Experimental results section establishes the supremacy of the hybrid domain watermarking technique over their single counterpart. This scheme is compared with some existing schemes in terms of various attacks and in terms of different watermarking embedding characteristics (as presented in table 20). This comparative analysis confirms the superiority of this proposed scheme.

**Table 20.** Comparative study of our proposed scheme with some existing schemes.

description	proposed scheme	Lai & Tsai [16]	Rastegar *et al*. [18]	Lagzian *et al*. [17]	Ganic & Eskicioglu [15]	Makbol & Khoo [12]
type of scheme	blind	blind	non-blind	non-blind	non-blind	blind
type of transform	DCT + SVD	DWT + SVD	Radon + DWT + SVD	RDWT + SVD	DWT + SVD	RDWT + SVD
watermark action before embedding	no action	no action	SVD	SVD	SVD	no action
size of host image	512 × 512	512 × 512	257 × 257	512 × 512	512 × 512	512 × 512
size of watermark image	256 × 256	256 × 256	33 × 33	512 × 512	256 × 256	512 × 512
tested host image	Lena, Pepper, Baboon, Barbara	Lena	Lena, Pepper and Baboon	Lena	Lena	Lena, Pepper and Baboon
type of watermark	Grey	Grey	Grey	Grey	Grey	Grey
False positive problem	no	yes	yes	yes	yes	yes

## Data accessibility

This article has no additional data.
